# Testing a Self-Determination Theory Model of Healthy Eating in a South African Township

**DOI:** 10.3389/fpsyg.2020.02181

**Published:** 2020-08-25

**Authors:** Jeroen De Man, Edwin Wouters, Peter Delobelle, Thandi Puoane, Meena Daivadanam, Pilvikki Absetz, Roy Remmen, Josefien van Olmen

**Affiliations:** ^1^Centre for General Practice, Department of Primary and Interdisciplinary Care, University of Antwerp, Antwerp, Belgium; ^2^Centre for Population, Family and Health, Department of Sociology, University of Antwerp, Antwerp, Belgium; ^3^School of Public Health, University of the Western Cape, Belville, South Africa; ^4^Chronic Disease Initiative for Africa, University of Cape Town, Cape Town, South Africa; ^5^Department of Public Health, Vrije Universiteit Brussel, Brussels, Belgium; ^6^Department of Food Studies, Nutrition and Dietetics, Uppsala University, Uppsala, Sweden; ^7^Health Systems and Policy Research Group, Department of Global Public Health, Karolinska Institutet, Stockholm, Sweden; ^8^International Maternal and Child Health Division, Department of Women’s and Children’s Health, Uppsala University, Uppsala, Sweden; ^9^Collaborative Care Systems Finland, Helsinki, Finland; ^10^Institute of Public Health and Clinical Nutrition, University of Eastern Finland, Kuopio, Finland; ^11^Department of Public Health, Institute of Tropical Medicine Antwerp, Antwerp, Belgium

**Keywords:** South Africa, type 2 diabetes, sub-Saharan Africa, healthy diet behavior, identified regulation, self-determination theory, introjected regulation, autonomous motivation

## Abstract

**Introduction:**

The burden of type 2 diabetes is growing rapidly in sub-Saharan Africa. Healthy eating has been shown to prevent the disease but is challenging to maintain. Self-determination theory offers a motivational framework for maintaining a healthy diet based on evidence from western settings. This study aims to assess whether self-determination theory can explain healthy diet behavior in a disadvantaged urban South African population.

**Methods:**

Cross-sectional data from a South African township population (*N* = 585; pre-diabetes = 292, diabetes = 293, age 30–75) were analyzed using structural equation modeling, while controlling for socio-demographic factors. Measures included self-reported autonomous and controlled motivation, perceived competence (measured through barrier self-efficacy), perceived relatedness (measured through perceived participation of significant others) and, as indicator for healthy diet, frequency of fruit, vegetable, and non-refined starch intake.

**Results:**

Healthy eating was positively associated (β = 0.26) with autonomous motivation, and negatively associated (β = −0.09) with controlled motivation. Perceived competence and relatedness were positively associated with healthy eating (β = 0.49 and 0.37) and autonomous motivation (β = 0.65 and 0.35), and negatively associated with controlled motivation (β = −0.26 and −0.15). Autonomous motivation mediated the effect of perceived competence and relatedness on healthy eating. The model supported a negative association between controlled and autonomous motivation.

**Conclusion:**

This is the first study providing evidence for self-determination theory explaining healthy eating in a disadvantaged sub-Saharan African setting among people at risk of or with diabetes type two. Our findings suggest that individuals who experience support from friends or family and who feel competent in adopting a healthy diet are more likely to become more motivated through identifying the health benefits of healthy eating as their goal. This type of autonomous motivation was associated with a healthier diet compared to individuals whose motivation originated in pressure from others or feelings of guilt or shame. Our recommendations for public health interventions include: focus on the promotion of diet-related health benefits people can identify with; encourage social support by friends or family; reinforce people’s sense of competence and skills; and avoid triggering perceived social pressure or feelings of guilt.

## Introduction

Type 2 diabetes (T2D) is one of the leading causes of death and disability, and its prevalence has been growing rapidly in sub-Saharan Africa (International Diabetes Federation, 2017). In response to this T2D pandemic, engaging in healthy lifestyle activities such as healthy eating, can substantially reduce the risk of T2D onset and complications (International Diabetes Federation, 2017). However, maintaining a healthy diet has been shown challenging and motivation is believed to be a crucial factor, also because the benefits are often not immediately apparent (Kwasnicka et al., 2019). Understanding the motivational dynamics of maintaining a healthy diet can help to support someone at the individual level, but it can also guide the design of interventions and policies at the public health level.

Self-Determination Theory (SDT) proposes a promising explanatory framework to predict self-regulated behavior which has been shown to be particularly relevant for dietary behavior (Verstuyf et al., 2012). Central to SDT is that motivation can differ in quality with a major distinction between autonomous (self-determined) and controlled motivation (Deci and Ryan, 2000). Controlled motivation occurs when one is regulated by sources external to the actual behavior such as incentives, perceived approval from others or avoidance of punishment (i.e., external regulation) (Deci and Ryan, 2000). Another form of controlled motivation occurs when one is regulated by partially internalized sources such as self-worth (pride) or threats of guilt and shame (i.e., introjected regulation) (Deci and Ryan, 2000). Individuals moved by controlled motivation are shown to quickly lose interest in pursuing their specific behavior once the external driver disappears (Deci and Ryan, 2000).

Autonomous motivation, in contrast, is more self-determined and emanates from within oneself, from personal interests, or from abiding values (Deci and Ryan, 2000). Compared to controlled motivation, autonomous motivation has been shown to lead to enhanced performance, persistence, and creativity (Deci and Ryan, 2000). Identified regulation is a form of autonomous motivation that occurs when someone is driven by personal goals or values they associate with a specific behavior. With regards to a healthy diet, an evident goal is to pursue ‘being healthy.’ Through this process of identification, individuals endorse their own behavior which has been shown to increase commitment and performance (Deci and Ryan, 2000). Identified regulation is deemed particularly relevant to dietary behavior since more intrinsic forms of autonomous motivation imply that the individual is driven by joy or pleasure, which may be less straightforward to derive from maintaining a healthy diet (Verstuyf et al., 2012).

Self-Determination Theory further argues that social context plays a crucial role in one’s motivation through the satisfaction or thwarting of the individual’s basic psychological needs: perceived competence, autonomy, and relatedness (Deci and Ryan, 2000). Perceived competence corresponds to one’s sense of efficacy toward a specific action with respect to their internal and external environment. Perceived autonomy corresponds to one’s sense of choice and volition in regulating their behavior. Perceived relatedness corresponds to their sense of support from significant others. Contexts that satisfy the individual’s basic psychological needs have been shown to foster autonomous motivation (Deci and Ryan, 2000), and hence more sustainable regulation. In contrast, thwarting those needs will hamper internalization of motivation (Deci and Ryan, 2000).

Research on SDT in the domain of eating regulation is still in its early stages (Verstuyf et al., 2012). Only a handful of studies have shown a positive association between more autonomous forms of motivation and a healthy diet in populations that may benefit from dietary behavior change (Verstuyf et al., 2012), including people with diabetes (Shigaki et al., 2010; Nouwen et al., 2011). More controlled motivation had no or a negative association with a healthy diet in those studies (Verstuyf et al., 2012). Even less studies have addressed the association between types of motivation and experiences of need satisfaction or thwarting (Verstuyf et al., 2012). Furthermore, recent studies in other domains including physical activity behavior have challenged the tenets of SDT. Those studies have shown that a combination of high controlled and autonomous forms of motivation can lead to better outcomes than high autonomous motivation alone (Langan et al., 2016). This mixed evidence emphasizes the need to study the relationship between both forms of motivation. Finally, research on SDT and diet has completely ignored the sub-Saharan African context. While evidence exists on the cross-cultural validity of SDT in other domains (Deci and Ryan, 2000), this evidence is lacking for diet which is known to be complex and contextually dependent.

In this study we use structural equation modeling (SEM) to test the validity of the major relationships between psychological constructs of the SDT framework and healthy eating while adjusting for potential non-psychological confounders (e.g., socio-demographic factors). The study focuses on people recently diagnosed with T2D or at high risk for T2D, because we believe that understanding motivational dynamics taking place in the early months following diagnosis is crucial for the promotion of dietary behavior change.

The study takes place in Khayelitsha, a peri-urban township in the Western Cape with an estimated population of 400.000, a population density of more than 10.000 people per km^2^, a high unemployment rate and low education attainment (Strategic Development Information and GIS Department City of Cape Town, 2011). Studies estimate the prevalence of obesity (BMI > 30 kg/m^2^) in this population to be higher than 50% for women and 18% for men (Malhotra et al., 2008). The high prevalence has primarily been linked to unhealthy dietary habits (Malhotra et al., 2008; Masupe et al., 2018). Easy access to ‘convenience food’ and social pressures based on traditional beliefs have been reported to negatively influence people’s diet, while healthier food is available and affordable (Masupe et al., 2018; De Man et al., 2019). We therefore think that studying the motivational dynamics in this context is important. Findings from this setting are not only deemed relevant for the millions of people living in similar settings in South Africa, but also for other Sub-Saharan African countries that have known and will know rapid urbanization.

**FIGURE 1:**
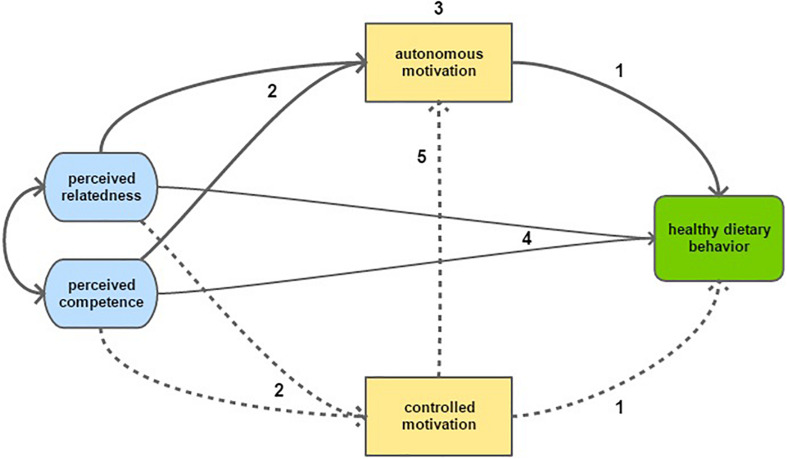
Graphical representation of the study hypotheses, including 2 of the 3 psychological needs: perceived competence and perceived relatedness. Solid arrows represent hypothesized positive associations, while dotted arrows represent hypothesized negative associations. Numbers relate to the hypotheses presented in the text.

We hypothesized the following relationships (see Figure 1): (1) a positive effect of autonomous motivation, and no effect of controlled motivation on healthy eating; (2) in line with SDT’s psychological needs theory, a positive effect of perceived relatedness and competence on autonomous motivation and no effect of perceived relatedness and competence on controlled motivation; (3) autonomous motivation partially mediates the effect of perceived competence and relatedness on healthy eating; (4) a total positive effect of perceived relatedness and competence on healthy eating; (5) a negative effect of controlled motivation on autonomous motivation. Although perceived autonomy is one of the three psychological needs, no appropriate measure for this context was available at the start of our study. In line with the SDT model proposed for other domains, we allow for a correlation between perceived relatedness and competence (Eriksson and Boman, 2018).

## Materials and Methods

### Study Design and Procedures

This study is part of the SMART2D trial, a cluster-randomized adaptive implementation trial aimed at improving self-management among people at risk of, or living with T2D (Guwatudde et al., 2018). The present study aims to validate part of the theory-driven framework that guided implementation of the SMART2D interventions (De Man et al., 2019). The study analyzed cross-sectional baseline data collected from SMART2D participants in Khayelitsha, a peri-urban township in the Western Cape in South Africa.

The 585 study participants were residents of the township for at least 6 months prior to enrollment, aged 30–75 years, not diagnosed with diabetes for longer than 12 months, having been diagnosed with or at risk of diabetes by a health care provider, and without serious mental disability. Eligible participants were recruited in two community health centers located in the township upon referral by a health care worker. A questionnaire was administered by trained field workers between August 2017 and November 2018. The questionnaire included socio-demographic items and diet- and motivation-related scales, complemented by anthropometric and biochemical measurements.

### Measures

The SDT concept of perceived competence was measured through barrier self-efficacy (or self-regulatory efficacy) which corresponds to the perceived capability to maintain a healthy diet given various conditions or impediments (i.e., barriers). This proxy measure was chosen because it is deemed to be adaptable to different, including non-western, contexts. Six items were proposed to assess self-belief in coping with a variety of barriers to maintaining a healthy diet as proposed by Schwarzer et al. (2007) and Hankonen (2011) (see Additional File 1). Two of the six items were ignored because of ill fit (correlated errors were high among those items). The items retain a common semantic structure: ‘Do you think you can do X, even if Y (barrier),’ for example: ‘Do you think you can maintain a healthy diet even if you are not used to the taste of these foods?’ Potential barriers were chosen based on their relevance to the study setting. Participants responded to each item on a five-point Likert-type scale ranging from 1 (strongly disagree) to 5 (strongly agree).

To measure the concept of perceived relatedness, a modified version of the scale for participation and involvement of family members and friends in diet behavior was used as a proxy measure. This scale was developed by Sallis et al. (1987) and has been used and validated in a variety of contexts. Five items were selected based on their cross-cultural relevance and factor loadings in previous studies (Sallis et al., 1987) (see Additional File 1). One item was ignored because of ill fit (correlated errors were high). The questions shared the stem: ‘How often have people close to you (friends, family or relatives)?,’ followed by items such as ‘eaten healthy food with you,’ ‘encouraged you to stick with your healthy diet’ etc. Possible responses included: ‘Never,’ ‘less than once a week,’ ‘once a week,’ and ‘more than once a week.’ The questions were introduced by the following statement to emphasize the idea of perceived social support: ‘We want to understand to what extent people close to you (friends, family, or relatives) have helped you to maintain a healthy diet.’

Autonomous and controlled motivation toward maintaining a healthy diet were assessed through the Treatment Self-Regulation Questionnaire (TSRQ) for people with diabetes. Validation studies (Levesque et al., 2007) and studies investigating dietary behavior among diabetes patients (Williams et al., 1998) report adequate reliability and validity of this questionnaire. Guided by factor loadings from a validation study by Levesque et al. (2007), four items were selected to measure autonomous motivation (i.e., identified regulation) and four items to measure controlled motivation (i.e., external and introjected regulation). Items belonging to different types of motivation were mixed in the questionnaire to decrease potential acquiescence bias (see Additional File 1). The items, in the TSRQ presented as statements, were transformed to questions asking why participants would maintain a healthy diet. An example of an item testing autonomous motivation: ‘Would you maintain a healthy diet because you feel that you want to take responsibility for your own health?’ and an example of an item testing controlled motivation: ‘Would you maintain a healthy diet because you would feel guilty or ashamed of yourself if you didn’t?’ Participants responded to each item on a five-point Likert-type scale ranging from 1 (strongly disagree) to 5 (strongly agree).

Healthy eating was measured through a construct of self-reported frequency of intake of fruit, vegetables, and non-refined starch. This measure was based on questions modified from the WHO STEPS survey (World Health Organization, 2017) with answer options 0 to 7 with a higher score being healthier. Participants were asked to think of what they usually eat: ‘In a typical week, how many days do you eat…?’ followed by the following specific food groups: ‘fruit,’ ‘vegetables,’ or ‘non-refined starch.’ Contextual examples of these food groups were included in the questions (see Additional File 1). The selection of the food groups was based on a combination of what the study participants perceive as healthy food (Muzigaba and Puoane, 2013; De Man et al., 2019) and evidence showing their protective effect against diabetes (de Munter et al., 2007; Fan et al., 2014).

To address potential confounding, covariates were added based on their assumed effect on the latent constructs. BMI and education were included for the relationship between motivational constructs (Verstuyf et al., 2012; Teixeira et al., 2015). Household income, education, BMI, marital status, age and sex were included for the relationship between motivational constructs and dietary behavior (Williams et al., 1998; Verstuyf et al., 2012; Teixeira et al., 2015) (see Table 1).

**TABLE 1:** Summary statistics of socio-demographic and dietary behavior characteristics of the study population (*N* = 585).

Demographic characteristics*	IQ-range OR N		Mean ± SD OR Proportion
Age (years)	44–59		51 ± 10
Monthly HH income (US$)**	154–385		302 ± 229
**Body Mass Index**			
(0–25)	61		10%
(25–30)	131		22%
(30–35)	143		24%
(35–40)	126		22%
(45–70)	124		21%
**Sex**			
Female	424		72%
Male	161		28%
**Education**			
None	15		3%
Primary grade < 5–7	137		23%
Secondary grades 8–10	191		33%
Secondary grades 11–12	218		37%
Higher	24		4%
**Civil status**			
Married or cohabiting	282		48%
Other	303		52%
**Employment**			
Yes	259		44%
No	326		56%
**Diagnosis**			
T2D	293		50%
At risk of T2D	292		50%

Dietary behavior ***	**Median**	**IQ-range**	**Missing (N)**

Fruit	3	2–6	4
Vegetables	5	3–7	1
Non-refined starch	4	2–7	5

*The second column presents the interquartile range for age and income, and total number for categorical variables. The third column presents mean and standard deviation for age and income, and proportion for categorical variables. **Household incomes were reported in South African Rand and converted to US dollar (13 ZAR = 1 USD). ***Intake frequency in days per week.

### Contextual Adaption

All measures were translated into the local language of the study population (i.e., isiXhosa), and adapted to the local context. Measures were back translated to English and adjustments made where necessary to ensure their meaning was kept. Local validity was ensured through piloting in a non-study area and training of data collectors (e.g., through mock interviews).

### Missing Data

The percentage of missing data varied between 0.0 and 1.0%, except for household income which was missing in 5.2% of cases. Multivariate imputation by chained equations with predictive mean matching was used to handle the missing data under a missing at random assumption. The procedure was done using the ‘Mice’ package in R. Rubin’s rules were used to pool point and SE estimates across 20 imputed data sets.

### Data Analysis

Data were analyzed with R software and the packages ‘lavaan’ and ‘semTools.’ Confirmatory factor analysis was used to test whether the item indicators of healthy eating, controlled and autonomous motivation, perceived competence, and perceived relatedness loaded adequately on the latent variables. The overall fit of the measurement model was tested using the comparative fit index (CFI), Tucker-Lewis index (TLI), and root mean square error of approximation (RMSEA) with a 90% confidence interval. Acceptable model fit was defined by the following thresholds: RMSEA (≤0.08), SRMR (≤0.08), CFI and TLI (≥0.95) (Hu and Bentler, 1999). Multiple indices were used to assess model fit more adequately. Latent constructs were assumed to follow normal distributions underlying observed categorical indicators treated as ordered categorical. Diagonally weighted least squares was used for parameter estimation. A second order model was used for controlled motivation since it included items of external and introjected regulation.

Structural equation modeling was used to test if the data fit the hypothesized SDT model. Model fit was assessed using measures of fit (CFI, TLI, RMSEA, and RSMR) and regressions or correlations between the latent constructs. As defined by Cerin (2010), criteria for mediation included: (1) the mediating variable is related to the independent variable; and (2) occurrence of a significant association between the mediating variable and the outcome variable, after adjustment for the independent variable. To account for the potential effect of covariates, multiple indicator, multiple cause models (MIMIC) were used. Based on a single input matrix combining variance and covariances of both latent variables and non-latent covariates, these models can control for confounding and effect modification. In addition, the relationship between controlled motivation on autonomous motivation was tested using the Satorra-Bentler scaled chi-square difference test for two nested models (Satorra and Bentler, 2001): (1) a model with the relationship constrained to zero, and (2) a model with autonomous motivation regressing on controlled motivation.

## Results

The study population had a higher proportion of females (72%), more than half was unemployed (56%), and about one quarter had not obtained a secondary educational degree (27%). Household income varied substantially with 48% of the study population living under the national poverty line of 60 USD per household member per month (Statistics South Africa, 2018). 90% of participants had a BMI > 25. Table 1 provides more detail about the background characteristics of study participants, including descriptive statistics of their dietary behavior.

### Measurement Models

For the motivational constructs, we assumed uncorrelated measurement errors and no double-loading indicators. Unstandardized factor loadings were all significant (z > 1.96) and the lowest standardized factor loading was 0.60 (for controlled motivation). For the constructs of perceived relatedness, perceived competence and autonomous motivation, CFI and TLI ranged from 0.99 to 1 for, SRMR from 0.005 to 0.046 and RMSEA from 0.00 to 0.081. The fully standardized factor loadings of the food construct (just identified) ranged from 0.531 to 0.596. Estimates of unstandardized and completely standardized factor loadings in the full (structural) model are reported in Additional File 2.

### Structural Model

**FIGURE 2:**
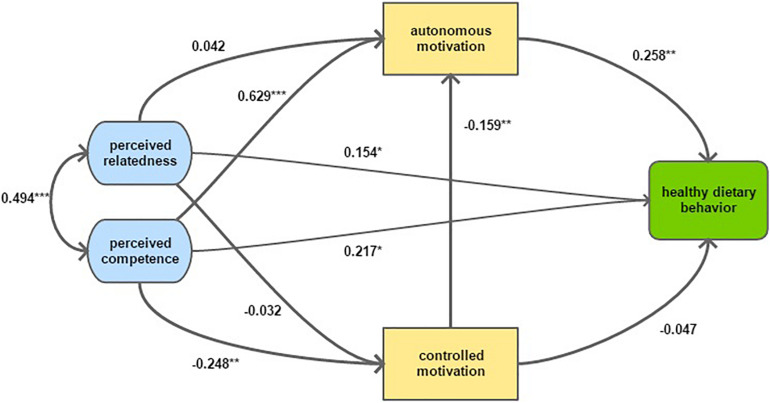
Structural model displaying direct effects between motivational constructs and dietary behavior. Parameter estimates are fully standardized. *p-value < 0.05; **p-value < 0.01; ***p-value < 0.005.

We tested the model as hypothesized in the introduction with perceived competence and relatedness allowed to correlate. Figure 2 displays the single effects of the structural model using completely standardized regression parameters. Table 2 presents the combined effects and the measures of fit of a model without and with exogenous covariates as described in the method section. Estimates of unstandardized regression parameters with corresponding standard errors and *p*-values are reported in Additional File 2.

**TABLE 2:** Combined effects (direct and indirect effects together) between motivational constructs and dietary behavior.

Combined effect			Baseline model	Adjusted model^$^
Autonomous motivation	→	Dietary behavior	0.260**	0.258**
Controlled motivation	→	Dietary behavior	−0.098*	−0.089*
Perceived competence	→	Autonomous motivation	0.676***	0.649***
Perceived relatedness	→	Autonomous motivation	0.374***	0.353***
Perceived competence	→	Controlled motivation	−0.294***	−0.264***
Perceived relatedness	→	Controlled motivation	−0.174***	−0.154**
Perceived competence	→	Dietary behavior	0.525***	0.485***
Perceived relatedness	→	Dietary behavior	0.409***	0.366***
**Model fit:**				
RMSEA			0.035	0.037
90% CI RMSEA			0.027– 0.042	0.032– 0.042
RSMR			0.050	0.050
CFI			0.995	0.987
TLI			0.994	0.993

Values represent fully standardized regression coefficients. *p-value < 0.05; **p < 0.01; ***p < 0.005. ^$^Motivational constructs adjusted for BMI and education; dietary behavior adjusted for household income, education, BMI, marital status, age, and sex.

Both models were identified and resulted in an acceptable fit. Comparison with a model without a unidirectional relationship from controlled to autonomous motivation showed a significantly lower chi-square value (*p*-value = 0.00) and an improvement of all measures of fit in favor of inclusion of the unidirectional relationship.

The final structural equation model displayed in Figure 2 indicated a direct positive effect of autonomous motivation, perceived competence and perceived relatedness on healthy eating. The model indicated a direct negative effect of controlled motivation on autonomous motivation. Furthermore, the model indicated a strong positive effect of perceived competence on autonomous motivation and a negative effect of perceived competence on controlled motivation. Both psychological needs (i.e., perceived competence and relatedness) are strongly correlated.

However, to fully appreciate the relationship between these constructs, combined effects (i.e., direct and indirect pathways taken together) were assessed (see Table 2).

Calculations of these combined effects confirmed a positive association between autonomous motivation and healthy eating and a negative association between controlled motivation and healthy eating. Perceived relatedness and competence both had a combined positive effect on autonomous motivation, a negative effect on controlled motivation and a positive effect on healthy eating.

The criteria of autonomous motivation being a mediator are fulfilled because of the positive associations between autonomous motivation and both perceived relatedness and competence, in combination with the positive association of autonomous motivation with healthy eating. Similarly, controlled motivation mediated a negative effect between perceived competence and relatedness and dietary behavior.

## Discussion

This study is the first in providing evidence for SDT in explaining healthy dietary behavior in an urban sub-Saharan African population at risk of or living with T2D. Our findings indicate a positive effect of autonomous motivation and a smaller negative effect of controlled motivation on dietary behavior. The results further support the role of perceived relatedness and competence as basic psychological needs because of their positive effect on autonomous motivation and dietary behavior. Finally, the model indicates a negative effect of controlled motivation on autonomous motivation. The excellent fit of the data to the hypothesized model supports these findings.

The positive association between autonomous motivation and healthy eating among people with or at risk of T2D in this study is in line with studies conducted in western settings (Sentcal et al., 2000; Williams et al., 2004; Shigaki et al., 2010; Nouwen et al., 2011). Evidence on the association between controlled motivation and healthy eating has been mixed. Some studies found no association in people with T2D (Julien et al., 2009), while other studies found a negative association (Pelletier et al., 2004). Interestingly, Pelletier et al. (2004) found that autonomous motivation was associated with what one eats or rather the quality of food, whereas controlled motivation was associated with the concern with how much one eats, or rather the quantity of food. Since our study was focused on what one eats, Pelletier’s findings may explain the more significant effect between the outcome and autonomous motivation versus controlled motivation. A strength of this study is that it controls for socio-economic factors including household income and education. Controlling for those factors did not lead to major changes in the relationship between motivational constructs and dietary behavior. While our findings do not challenge the importance of such socio-economic factors in the prediction of dietary behavior, they emphasize the importance of motivational factors.

Perceived competence (measured as barrier self-efficacy) and perceived relatedness (measured as perceived participation of significant others) both showed a strong association with healthy eating, which is in line with studies in western settings (Sentcal et al., 2000; Williams et al., 2004; Anderson et al., 2007; King et al., 2010; Nouwen et al., 2011). Our model indicated a strong positive effect of perceived competence on autonomous motivation, and a negative effect on controlled motivation. Consequently, our model supports a mediating role of autonomous motivation between perceived competence and dietary behavior. For perceived relatedness, the total effect on both types of motivation followed the same trend but was substantially weaker. Moreover, as we allowed perceived competence and relatedness to correlate, this effect was almost fully mediated through perceived competence.

The findings provide evidence for SDT’s basic needs theory which posits that satisfaction of perceived competence and relatedness fosters autonomous motivation. For dietary behavior, we did not find any previous studies addressing these links, but our findings are in line with evidence on other types of healthy behavior such as physical activity (Teixeira et al., 2012). The relatively weaker link between perceived relatedness and autonomous motivation is in line with empirical findings from other domains (Deci and Ryan, 2000), while for physical activity no consistent link was found between both concepts (Teixeira et al., 2012). In the light of those previous studies, our model suggesting a mediating role of perceived competence could be an indication that for diet, perceived relatedness would rather function as a condition to foster perceived competence, than as an independent basic need. Lastly, the measure of perceived relatedness used in this study focused on the frequency of participation of significant others. This measure was based on a widely used and validated scale, and as a measure of frequency, it was estimated to be more objective. However, it may have underestimated the actual perception of support participants experienced from significant others.

When testing for the relationship between both types of motivation, we found a better fit of a model that allows for a negative effect from controlled motivation to autonomous motivation. This finding suggests that a higher controlled motivation does not only lead to poor performance, but also may hamper the adoption of autonomous motivation. However, we should be careful in interpreting this finding, since a higher controlled motivation would be the result of the individual being exposed to a more controlling environment. This implies that controlled motivation does not necessarily have a direct negative effect on autonomous motivation, but rather that a controlling social environment would foster controlled motivation instead of autonomous motivation. Or, in SDT terms, a social environment thwarting people’s sense of volition and initiative will hamper the process of internalization of extrinsic motivation (Deci and Ryan, 2000). Different hypotheses have emerged about this matter and some recent studies in the field of physical activity show that coexistence of both controlled and autonomous motivation may improve outcome performance which in a way refutes the initial SDT hypothesis (Langan et al., 2016). While our study does not have the proper design to confirm either of those hypotheses, the finding of a negative relationship between controlled and autonomous motivation is in line with Ryan and Deci’s initial hypothesis (Deci and Ryan, 2000). As such, this finding suggests a need for further research on the effect of external triggers on one’s motivation (e.g., social pressure, advertisement, etc.). We think that such research is particularly needed in a setting were people are strongly influenced by aggressive marketing by the food industry and have easy access to unhealthy ‘convenience food’ (Masupe et al., 2018).

The exact definition of healthy eating is complex and has been a subject to debate. The variables selected in this study concern the frequency of intake of food items that have been shown healthy based on evidence, specifically fruits, vegetables, and non-refined starch. However, our construct is not exhaustive, and we did not include food items one should avoid in a healthy diet, such as sugar-sweetened beverages or refined starches. While we are not aware of studies comparing those variables with regards to SDT, this choice may have influenced our findings. Furthermore, variables focused on the frequency of intake, which may have influenced our findings as discussed above.

The findings from this urban sub-Saharan African context suggest that policies, interventions or health workers aiming to increase healthy eating will be more successful if they succeed in enhancing people’s willingness to take responsibility over their health. This can be done by emphasizing health-related benefits of healthy eating and through the establishment of a conducive social environment in which people feel genuinely supported and empowered. Furthermore, our findings indicate that reinforcing external triggers, such as social pressure, or introjected regulation (e.g., by eliciting feelings of guilt or shame) will likely thwart healthy eating behavior. Health workers and health programs should therefore avoid triggering such experiences.

### Limitations

Because of its cross-sectional design, this study does not allow to evaluate changes over time. Motivation is prone to change, and the patterns found in this study may not apply to people with long-standing diabetes. For example, the confrontation with limited improvements despite well-intended efforts may have a serious impact on how people are motivated. We need studies that capture and explain such changes over time. In addition, to show causality and evaluate effectiveness of interventions, intervention trials are needed, especially in low- and middle-income countries. Finally, the findings in our study context correspond to findings in western contexts, which supports the claim that SDT is cross-culturally valid. However, to fully confirm that the model is similar in different contexts, multi-country studies are needed using adequate statistical techniques (e.g., multiple-group structural equations).

Since our study relied exclusively on self-reported measures, shared method variance between predictor and outcome measures may have led to an overestimation of the strength of the relationship between the two measures (Doty and Glick, 1998). Self-reported measures are also subject to recall and social desirability bias.

## Conclusion

We found that people who report their diet to be more regulated based on their own choice of being healthy generally report a higher frequency of fruit, vegetable, and non-starch intake. On the other hand, people who report to be more pressured by their interpersonal environment (external regulation) or by feelings of guilt or shame (introjected regulation) report a lower intake of those food items. These findings support SDT’s claim that different types of motivation differ in quality based on their degree of self-determination (Deci and Ryan, 2000). Based on these findings, recommendations can be made for different levels of society. At the individual level, health workers and relatives or friends of people with T2D should offer support in fostering ownership, provide positive reinforcement of their competences, and show empathy and ‘be there for them.’ At the health policy level, interventions are likely to be more successful when they stimulate people to endorse their own choice and actions and when they strengthen people’s competences in pursuing a healthy diet. On the other hand, interventions triggering external or internal pressures are unlikely to help people in adopting a healthy diet and may even hinder them in doing so.

## Data Availability Statement

The datasets generated for this study are available on request to the corresponding author.

## Ethics Statement

The studies involving human participants were reviewed and approved by University of the Western Cape Biomedical Science Research Ethics Committee (BM17/1/36) and the Western Cape Government Department of Health. The patients/participants provided their written informed consent to participate in this study.

## Author Contributions

PA, MD, JO, TP, PD, and JD played a major role in the conception of the study. PA, MD, TP, PD, and JO played a major role in the design of the study. PD and TP were in charge of the data acquisition. JD drafted the manuscript and analyzed and interpreted the data. PA, MD, PD, EW, and RR substantially contributed to the interpretation of the data. All authors critically revised the manuscript for important intellectual content, and read and approved the final version.

## Conflict of Interest

The authors declare that the research was conducted in the absence of any commercial or financial relationships that could be construed as a potential conflict of interest.

## Abbreviations

BMI: Body Mass Index
NCDs: non-communicable diseases
SDT: Self-Determination Theory
T2D: type 2 diabetes.
