# Supplementary material for: Testing a Self-Determination Theory Model of Healthy Eating in a South African Township
Source: Front Psychol. 2020 Aug 25;11:2181. doi: 10.3389/fpsyg.2020.02181 (PMC7477942; doi:10.3389/fpsyg.2020.02181)
Supplement: Supplementary file 1 [file Table_1.DOCX]

# Additional file 1: Survey Questions

| BEHAVIOURAL MEASURES – HEALTHY DIET |
| --- |
| The next questions ask about some types of food that you usually eat. |
| In a typical week, on how many days do you eat **fruit**? |
| In a typical week, on how many days do you eat **vegetables** like tomatoes, carrots, onions, etc. (excluding tubers and high starch vegetables such as cassava, potatoes, matoke, yams, sweet potatoes)? |
| In a typical week, how many days do you eat **non-refined starch** (such as brown rice, whole grain pasta, wholegrain cereal, samp or whole meal/whole wheat / brown breads)? |

| PERCEIVED RELATEDNESS |
| --- |
| Intro: **We want to understand to what extent people close to you (friends, family or relatives) have helped you to maintain a healthy diet.** |
| How often have people close to you (friends, family or relatives) **eaten healthy food with you**? |
| How often have people close to you (friends, family or relatives) **encouraged you to stick with your healthy diet**? |
| How often have people close to you (friends, family or relatives) **changed their eating habits into healthier so you could eat the same foods**? |
| How often have people close to you (friends, family or relatives) **discussed healthy eating with you**? |
| How often have people close to you (friends, family or relatives) **helped you with eating healthy food on special occasions such as holidays, feasts, family gatherings**? |
|  |
| Answer options:  1= Never  2= less than once a week  3= once a week  4= more than once a week  888= no answer/not applicable |

| PERCEIVED COMPETENCE |
| --- |
| Intro: **We want to know if you can maintain a healthy diet under specific circumstances.** |
| Do you think you can maintain a healthy diet **even if you need to change how you cook at home**? |
| Do you think you can maintain a healthy diet **even if you are not used to the taste of these foods**? |
| Do you think you can maintain a healthy diet **even if this means that you have to eat less?** |
| Do you think you can maintain a healthy diet **even if your family or friends don’t help you**? |
| Do you think you can maintain a healthy diet **even if you would have to pay more for it**? |
| Do you think you can maintain a healthy diet **even if others around you eat unhealthy food**? |
| Answer options:  1 Strongly disagree  2 Disagree  3 Neutral  4 Agree  5 Strongly agree  888 Not applicable |

| AUTONOMOUS AND CONTROLLED MOTIVATION |
| --- |
| Intro: Think for a moment about the **reasons** why you actually would follow a healthy diet: why would you do this?  (*Instructions: give the patient a couple of seconds to think about it*).  We will now present possible reasons why you may follow a healthy diet. Please agree or disagree, using the options provided. |
| Would you maintain a healthy diet **because** **you personally believe it is the best thing for your health**? |
| Would you maintain a healthy diet **because** **you'd feel bad about yourself if you didn't**? |
| Would you maintain a healthy diet **because** **you feel pressure from others to do it**? |
| Would you maintain a healthy diet **because** **others would be upset with you if you didn't**? |
| Would you maintain a healthy diet **because it is very important for being as healthy as possible**? |
| Would you maintain a healthy diet **because** **you would feel guilty or ashamed of yourself if you didn’t**? |
| Would you maintain a healthy diet **because you feel that you want to take responsibility for your own health**? |
| Would you maintain a healthy diet **because** **it is an important choice you really want to make**? |
| Answer options:  1 Strongly disagree  2 Disagree  3 Neutral  4 Agree  5 Strongly agree  888 Not applicable |
