# Supplementary material for: Testing a Self-Determination Theory Model of Healthy Eating in a South African Township
Source: Front Psychol. 2020 Aug 25;11:2181. doi: 10.3389/fpsyg.2020.02181 (PMC7477942; doi:10.3389/fpsyg.2020.02181)
Supplement: Supplementary file 2 [file Table_2.DOCX]

# Additional file 2: Estimates of factor loadings and regression parameters

## Baseline model without covariates

Latent Variables:

Estimate Std.Err t-value df P(>|t|) Std.lv Std.all

D =~

vegF 1.000 0.663 0.663

fruitF 0.942 0.109 8.629 Inf 0.000 0.625 0.625

nrsF 0.624 0.095 6.540 Inf 0.000 0.414 0.414

AM =~

srn1 1.000 0.812 0.812

srn5 0.918 0.046 20.155 Inf 0.000 0.746 0.746

srn7 0.972 0.043 22.401 Inf 0.000 0.789 0.789

srn8 0.987 0.044 22.442 Inf 0.000 0.802 0.802

CM =~

CM1 1.000 0.470 0.470

CM2 3.145 0.704 4.468 Inf 0.000 1.327 1.327

CM1 =~

srn2 1.000 0.805 0.805

srn6 1.049 0.081 12.896 Inf 0.000 0.844 0.844

CM2 =~

srn3 1.000 0.897 0.897

srn4 0.974 0.048 20.314 Inf 0.000 0.874 0.874

PR =~

ssn1 1.000 0.948 0.948

ssn2 0.831 0.034 24.308 Inf 0.000 0.788 0.788

ssn3 0.886 0.033 27.025 Inf 0.000 0.840 0.840

ssn5 0.627 0.041 15.289 Inf 0.000 0.594 0.594

PC =~

effn2 1.000 0.846 0.846

effn3 1.003 0.029 34.088 Inf 0.000 0.849 0.849

effn4 0.985 0.029 33.674 Inf 0.000 0.834 0.834

effn6 0.871 0.032 27.411 Inf 0.000 0.737 0.737

Regressions:

Estimate Std.Err t-value df P(>|t|) Std.lv Std.all

D ~

AM (a) 0.212 0.084 2.515 Inf 0.012 0.260 0.260

CM (b) -0.098 0.082 -1.192 Inf 0.233 -0.056 -0.056

PC (c) 0.180 0.079 2.272 Inf 0.023 0.229 0.229

PR (d) 0.124 0.050 2.507 Inf 0.012 0.177 0.177

AM ~

PC (e) 0.631 0.046 13.808 Inf 0.000 0.657 0.657

PR (f) 0.032 0.045 0.718 Inf 0.473 0.037 0.037

CM ~

PC (g) -0.124 0.036 -3.460 Inf 0.001 -0.278 -0.278

PR (h) -0.012 0.020 -0.624 Inf 0.532 -0.031 -0.031

AM ~

CM (j) -0.351 0.069 -5.084 Inf 0.000 -0.163 -0.163

Covariances:

Estimate Std.Err t-value df P(>|t|) Std.lv Std.all

PR ~~

PC (i) 0.411 0.034 12.140 Inf 0.000 0.513 0.513

Defined Parameters:

Estimate Std.Err t-value df P(>|t|) Std.lv Std.all

totalAMtoD 0.212 0.084 2.515 Inf 0.012 0.260 0.260

totalCMtoD -0.173 0.081 -2.142 Inf 0.032 -0.098 -0.098

totalPRtoD 0.271 0.043 6.257 Inf 0.000 0.409 0.409

totalPCtoD 0.390 0.047 8.381 Inf 0.000 0.525 0.525

totalPRtoAM 0.291 0.040 7.283 Inf 0.000 0.374 0.374

totalPCtoAM 0.644 0.037 17.432 Inf 0.000 0.676 0.676

totalPRtoCM -0.064 0.022 -2.899 Inf 0.004 -0.174 -0.174

totalPCtoCM -0.129 0.035 -3.729 Inf 0.000 -0.294 -0.294

Factor loadings are reported under “Latent variables”, single effects are reported under “Regressions” and total effects (direct and indirect combined) are reported under “Defined Parameters”. In the first five columns: unstandardized parameters (estimate) with their standard errors (std.Err), related t-values and p-values. In the last two columns: the parameters with only the latent variable standardized (Std.lv) and completely standardized (Std.all) parameters. PR = perceived relatedness, PC= perceived competence, AM = autonomous motivation, CM = controlled motivation, D= Dietary behavior, vegF = weekly frequency of vegetable intake, fruitF = weekly frequency of fruit intake, nrsF = weekly frequency of non-refined starch intake, sex = gender, educ = level of education, mar = marital status, hhinc = household income

## Adjusted model with covariates

Latent Variables:

Estimate Std.Err t-value df P(>|t|) Std.lv Std.all

D =~

vegF 1.000 0.679 0.671

fruitF 0.963 0.115 8.380 Inf 0.000 0.653 0.646

nrsF 0.605 0.095 6.380 Inf 0.000 0.411 0.409

AM =~

srn1 1.000 0.822 0.809

srn5 0.931 0.049 19.074 Inf 0.000 0.765 0.755

srn7 1.000 0.047 21.259 Inf 0.000 0.821 0.809

srn8 1.000 0.048 20.877 Inf 0.000 0.821 0.809

CM =~

CM1 1.000 0.490 0.490

CM2 3.038 0.699 4.348 Inf 0.000 1.318 1.318

CM1 =~

srn2 1.000 0.810 0.807

srn6 1.034 0.076 13.522 Inf 0.000 0.837 0.834

CM2 =~

srn3 1.000 0.915 0.891

srn4 0.999 0.047 21.218 Inf 0.000 0.914 0.890

PR =~

ssn1 1.000 0.950 0.950

ssn2 0.828 0.035 23.933 Inf 0.000 0.787 0.787

ssn3 0.876 0.031 28.089 Inf 0.000 0.833 0.833

ssn5 0.629 0.041 15.455 Inf 0.000 0.598 0.598

PC =~

effn2 1.000 0.834 0.834

effn3 1.011 0.031 32.392 Inf 0.000 0.843 0.843

effn4 0.997 0.031 32.529 Inf 0.000 0.831 0.831

effn6 0.874 0.034 25.821 Inf 0.000 0.729 0.729

Regressions:

Estimate Std.Err t-value df P(>|t|) Std.lv Std.all

D ~

AM (a) 0.213 0.082 2.604 Inf 0.009 0.258 0.258

AM (b) -0.081 0.077 -1.046 Inf 0.296 -0.047 -0.047

PC (c) 0.177 0.077 2.306 Inf 0.021 0.217 0.217

PR (d) 0.110 0.048 2.310 Inf 0.021 0.154 0.154

AM ~

PC (e) 0.620 0.045 13.691 Inf 0.000 0.629 0.629

PR (f) 0.036 0.043 0.842 Inf 0.400 0.042 0.042

AM ~

PC (g) -0.118 0.036 -3.301 Inf 0.001 -0.248 -0.248

PR (h) -0.013 0.020 -0.655 Inf 0.512 -0.032 -0.032

AM ~

AM (j) -0.337 0.066 -5.077 Inf 0.000 -0.163 -0.163

D ~

hhinc 0.048 0.042 1.155 678.611 0.249 0.071 0.073

BMI 0.004 0.005 0.751 Inf 0.453 0.006 0.044

age 0.011 0.004 2.939 Inf 0.003 0.016 0.169

AM ~

BMI -0.001 0.002 -0.506 Inf 0.613 -0.003 -0.021

AM ~

BMI 0.003 0.006 0.530 Inf 0.596 0.004 0.030

D ~

educ_1 -0.038 0.312 -0.123 Inf 0.902 -0.056 -0.009

educ_2 -0.343 0.225 -1.525 Inf 0.127 -0.505 -0.140

educ_3 -0.304 0.206 -1.473 Inf 0.141 -0.447 -0.160

educ_4 -0.216 0.185 -1.169 Inf 0.243 -0.318 -0.149

educ_5 -0.162 0.179 -0.909 Inf 0.364 -0.239 -0.116

sex 0.044 0.096 0.462 Inf 0.644 0.065 0.029

mar 0.019 0.078 0.244 Inf 0.807 0.028 0.014

AM ~

educ_1 -0.351 0.295 -1.187 Inf 0.235 -0.427 -0.068

educ_2 -0.579 0.249 -2.322 Inf 0.020 -0.705 -0.195

educ_3 -0.315 0.226 -1.391 Inf 0.164 -0.383 -0.137

educ_4 -0.134 0.205 -0.652 Inf 0.514 -0.163 -0.077

educ_5 -0.071 0.198 -0.361 Inf 0.718 -0.087 -0.042

CM ~

educ_1 0.462 0.148 3.118 Inf 0.002 1.165 0.184

educ_2 0.334 0.124 2.687 Inf 0.007 0.841 0.233

educ_3 0.273 0.108 2.523 Inf 0.012 0.686 0.246

educ_4 0.217 0.095 2.285 Inf 0.022 0.548 0.257

educ_5 0.166 0.089 1.854 Inf 0.064 0.417 0.202

Covariances:

Estimate Std.Err t-value df P(>|t|) Std.lv Std.all

PR ~~

PC (i) 0.392 0.034 11.608 Inf 0.000 0.494 0.494

Defined Parameters:

Estimate Std.Err t-value df P(>|t|) Std.lv Std.all

totalAMtoD 0.213 0.082 2.604 Inf 0.009 0.258 0.258

totalCMtoD -0.153 0.076 -2.010 Inf 0.044 -0.089 -0.089

totalPRtoD 0.248 0.042 5.944 Inf 0.000 0.366 0.366

totalPCtoD 0.374 0.047 7.901 Inf 0.000 0.485 0.485

totalPRtoAM 0.279 0.040 6.946 Inf 0.000 0.353 0.353

totalPCtoAM 0.634 0.038 16.637 Inf 0.000 0.649 0.649

totalPRtoCM -0.059 0.022 -2.728 Inf 0.006 -0.154 -0.154

totalPCtoCM -0.123 0.035 -3.567 Inf 0.000 -0.264 -0.264

Factor loadings are reported under “Latent variables”, single effects are reported under “Regressions” and total effects (direct and indirect combined) are reported under “Defined Parameters”. In the first five columns: unstandardized parameters (estimate) with their standard errors (std.Err), related t-values and p-values. In the last two columns: the parameters with only the latent variable standardized (Std.lv) and completely standardized (Std.all) parameters. PR = perceived relatedness, PC= perceived competence, AM = autonomous motivation, CM = controlled motivation, D= Dietary behavior, vegF = weekly frequency of vegetable intake, fruitF = weekly frequency of fruit intake, nrsF = weekly frequency of non-refined starch intake, sex = gender, educ = level of education, mar = marital status, hhinc = household income.
